# Supplementary material for: Concentration-dependent effect of plant secondary metabolites on bacterial and fungal microbiomes in caterpillar guts
Source: Microbiol Spectr. 2023 Nov 22;12(1):e02994-23. doi: 10.1128/spectrum.02994-23 (PMC10783044; doi:10.1128/spectrum.02994-23)
Supplement: Figures S1 to S11, Table S1, S4 to S8 — Supplemental figures and tables. [file spectrum.02994-23-s0001.docx]

**Supplementary Information for**

Title: Concentration-dependent effect of plant secondary metabolites on bacterial and fungal microbiomes in caterpillar guts

Authors: Hana Šigutová, Petr Pyszko, Martin Šigut, Kateřina Czajová, Martin Kostovčík, Miroslav Kolařík, Denisa Hařovská, Pavel Drozd

**This file includes:**

Figures S1 to S11

Tables S1, S4–S8

**Other supplementary materials for this manuscript are uploaded separately, and include the following:**

Table S2 (contaminant bacterial and fungal ASVs)

Table S3 (bacterial and fungal taxa, the number of reads, and the variables entering the analyses)

**Fig. S1.** Results of qPCR analysis. Boxplots showing the difference of bacterial and fungal loads in caterpillar guts (number of cells/g of gut tissue) across a) treatment types (PSM types: salicylic acid, tannic acid, tannivin; Control: caterpillars fed by leaves, fed by AD without PSM or starved individuals) and b) PSM concentration (low, medium or high)

**
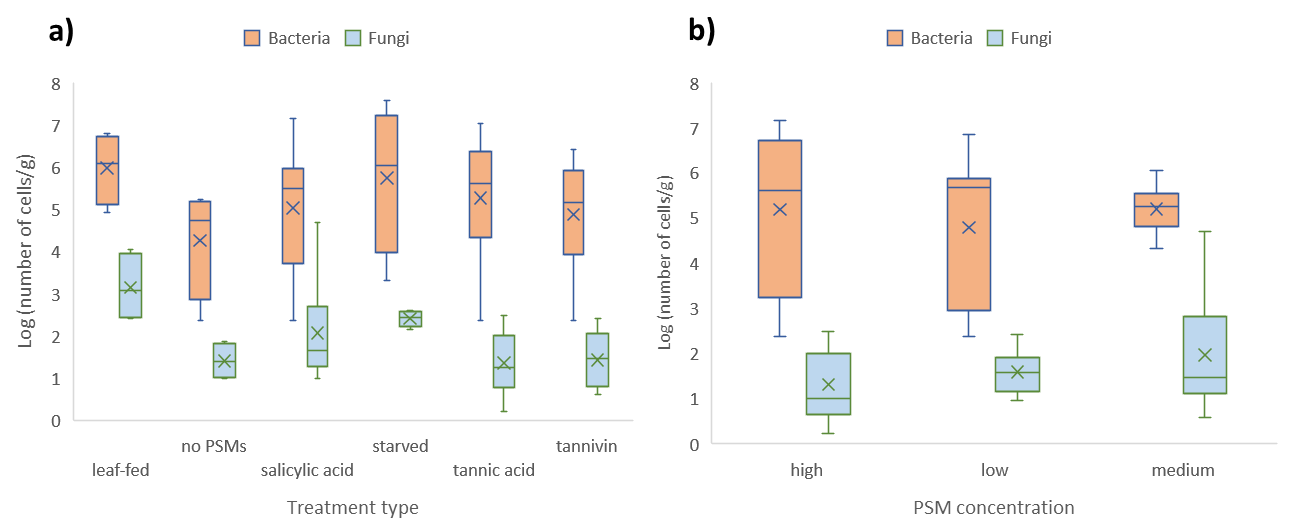
**

**Fig. S2.** Ratio between initial and final weight for AD-fed caterpillars in relation to compound types and concentration levels for individual species: a) *Agriopis aurantiaria*, b) *Catocala sponsa*, c) *Erannis defoliaria*, d) *Eupsilia transversa*, e) *Hypaurotis quercus*, f) *Lymantria dispar*, g) *Orthosia cruda*, h) *Orthosia miniosa*, i) *Phigalia pilosaria* (mean ± SE). Individual letters indicate groups that are significantly different from each other according to the main factor displayed on x-axis (Diet type)


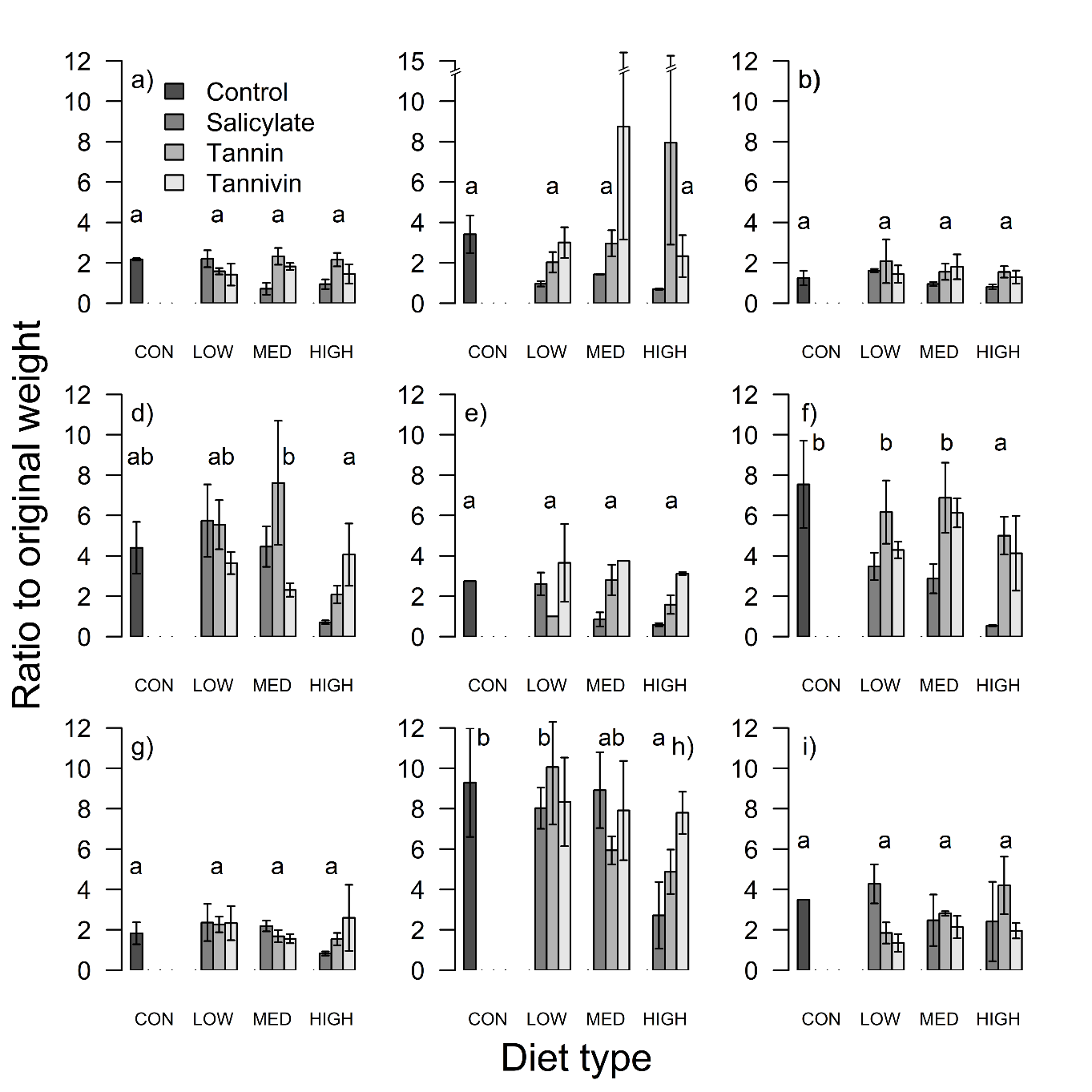


**Fig. S3.** Number of reads ranged between 884 and 69047 for bacteria (95%; mean = 17588.95 ± SE 945.20 for larvae; 6926.30±1264.56 for diet), and between 30 and 28517 for fungi (95%; 3796.58 ± 463.61 for larvae; 3391.33±997.74 for diet). Rarefaction curves for individual samples for a) bacterial genera and b) fungal species. The rarefactions reaching mostly asymptotes show that the sequential depth was sufficient.


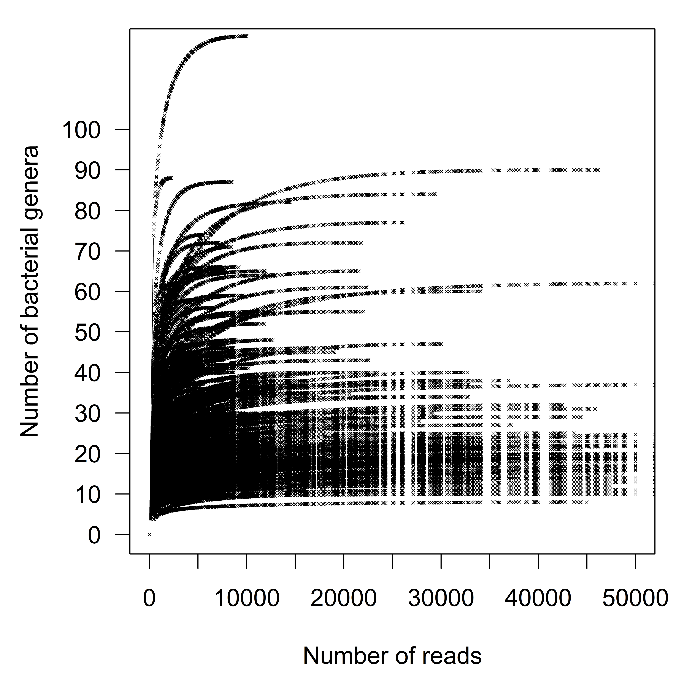

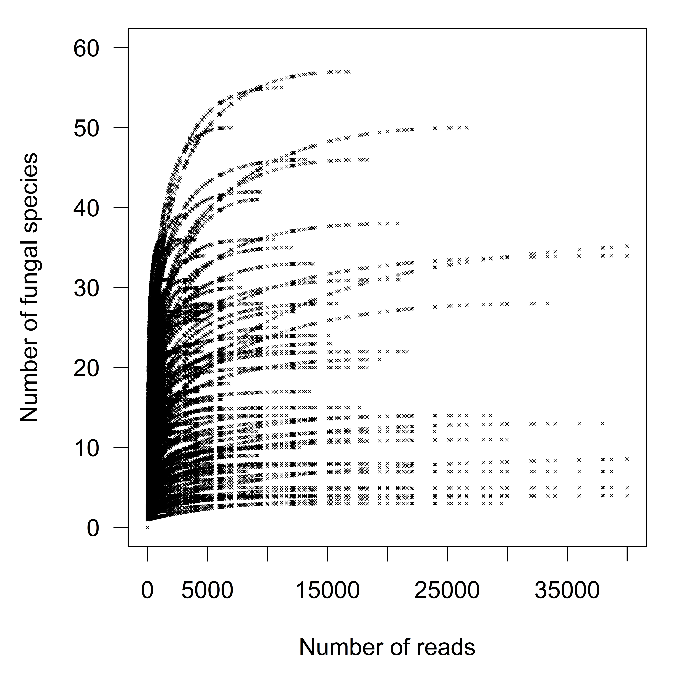


**Fig. S4**. Differences in rarefied bacterial genera richness in relation to individual concentration levels and compound types for individual species: a) *Agriopis aurantiaria*, b) *Catocala sponsa*, c) *Erannis defoliaria*, d) *Eupsilia transversa*, e) *Hypaurotis quercus*, f) *Lymantria dispar*, g) *Orthosia cruda*, h) *Orthosia miniosa*, i) *Phigalia pilosaria* (mean ± SE). Individual letters indicate groups that are significantly different from each other according to the main factor displayed on x-axis (Diet type)


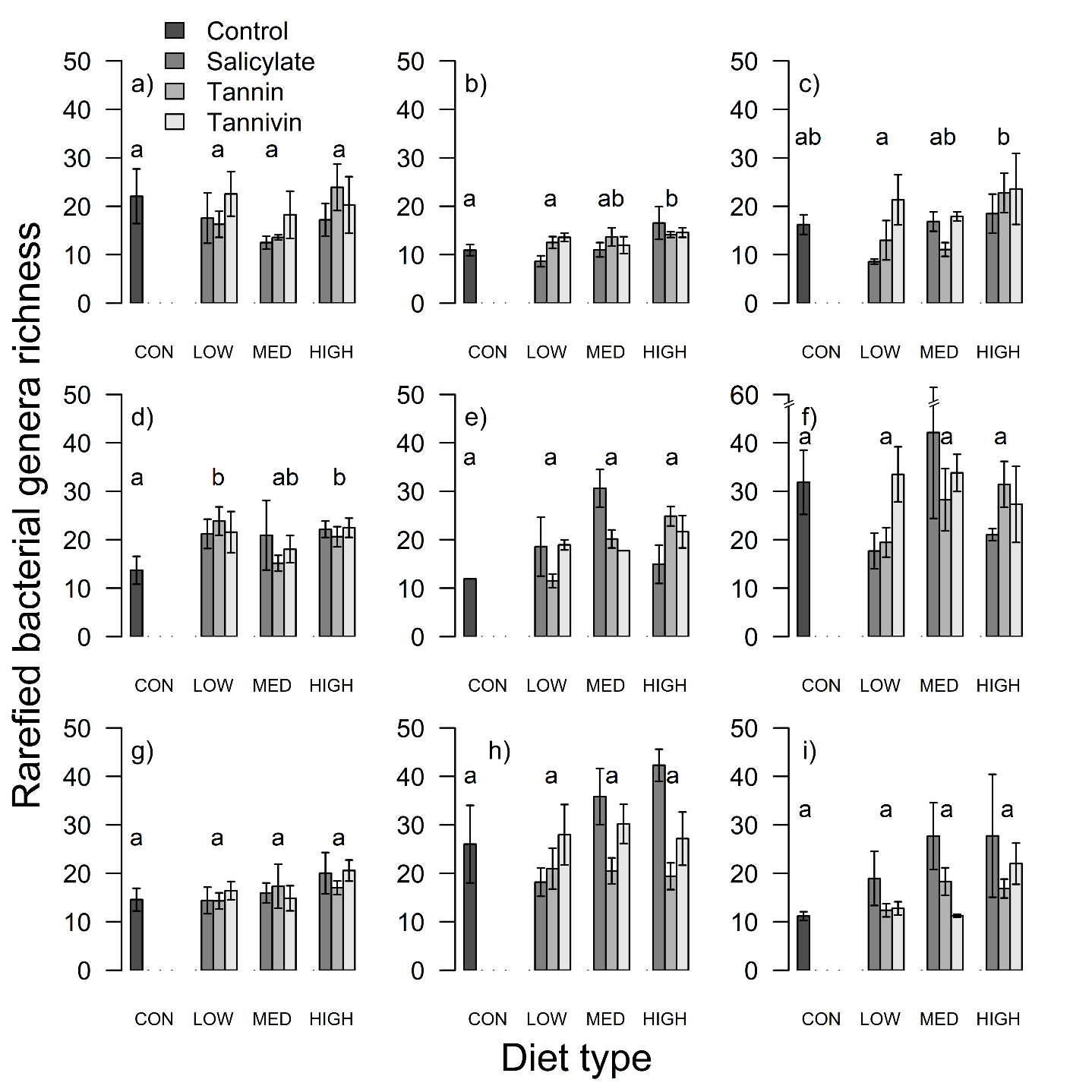


**Fig. S5**. Differences in rarefied fungal species richness in relation to individual concentration levels and compound types for individual species: a) *Agriopis aurantiaria*, b) *Catocala sponsa*, c) *Erannis defoliaria*, d) *Eupsilia transversa*, e) *Hypaurotis quercus*, f) *Lymantria dispar*, g) *Orthosia cruda*, h) *Orthosia miniosa*, i) *Phigalia pilosaria* (mean ± SE). Individual letters indicate groups that are significantly different from each other according to the main factor displayed on x-axis (Diet type)


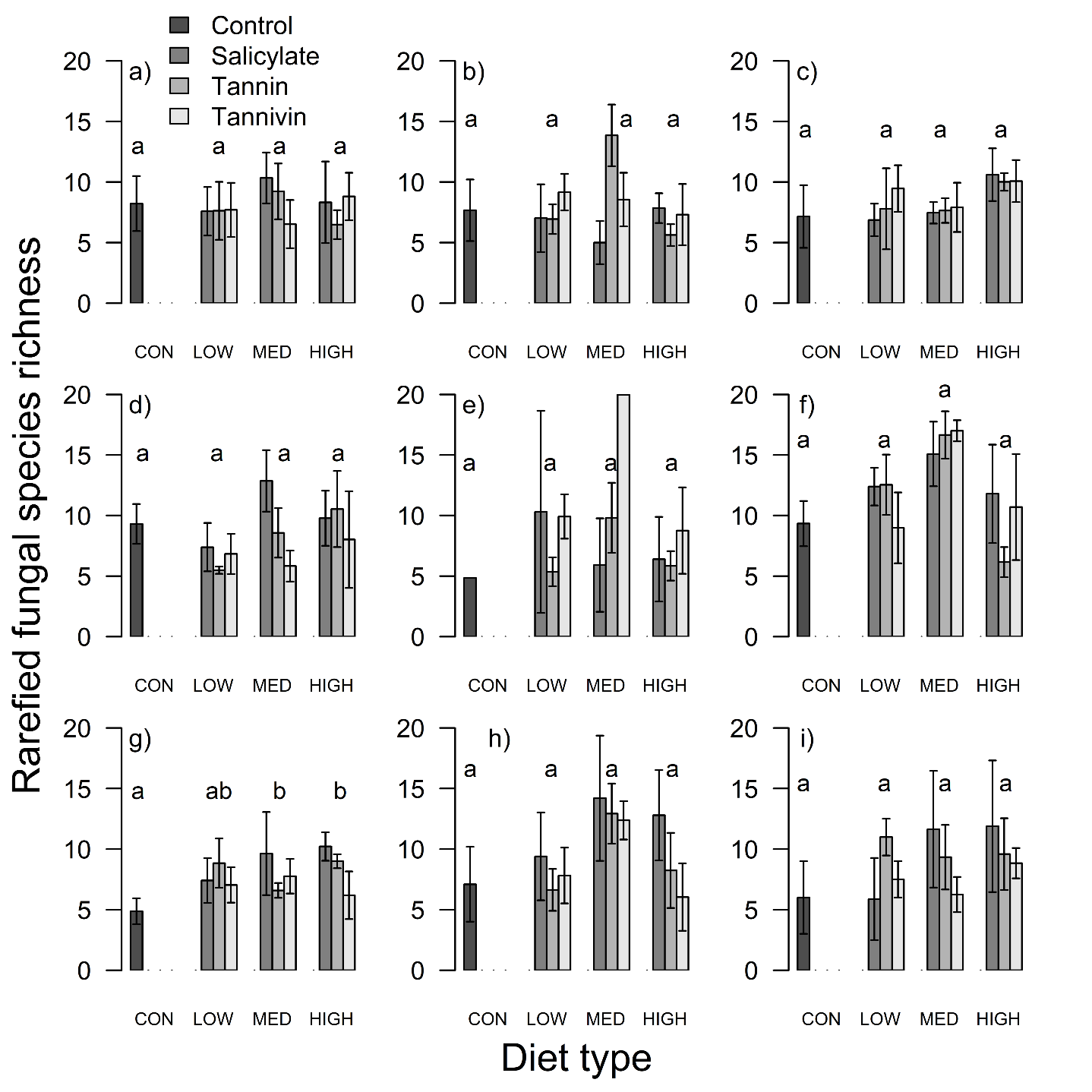


**Fig. S6.** In artificial diet, bacterial richness was explainable neither by fungal richness (df = 28, F = 1.34, P = 0.258), nor concentration level (df = 25, F = 0.45, P = 0.722), or compound type (df = 23, F = 0.21, P = 0.810). Similarly, fungal richness was explainable neither by bacterial richness (df = 28, F = 1.49, P = 0.233), nor concentration level (df = 25, F = 1.11, P = 0.365), or compound type (df = 23, F = 0.61, P = 0.549). The figure shows the differences in rarefied richness found in artificial diet in relation to individual concentration levels and compound types for a) bacterial genera and b) fungal species (mean ± SE). Individual letters indicate groups that are significantly different from each other according to the main factor displayed on x-axis


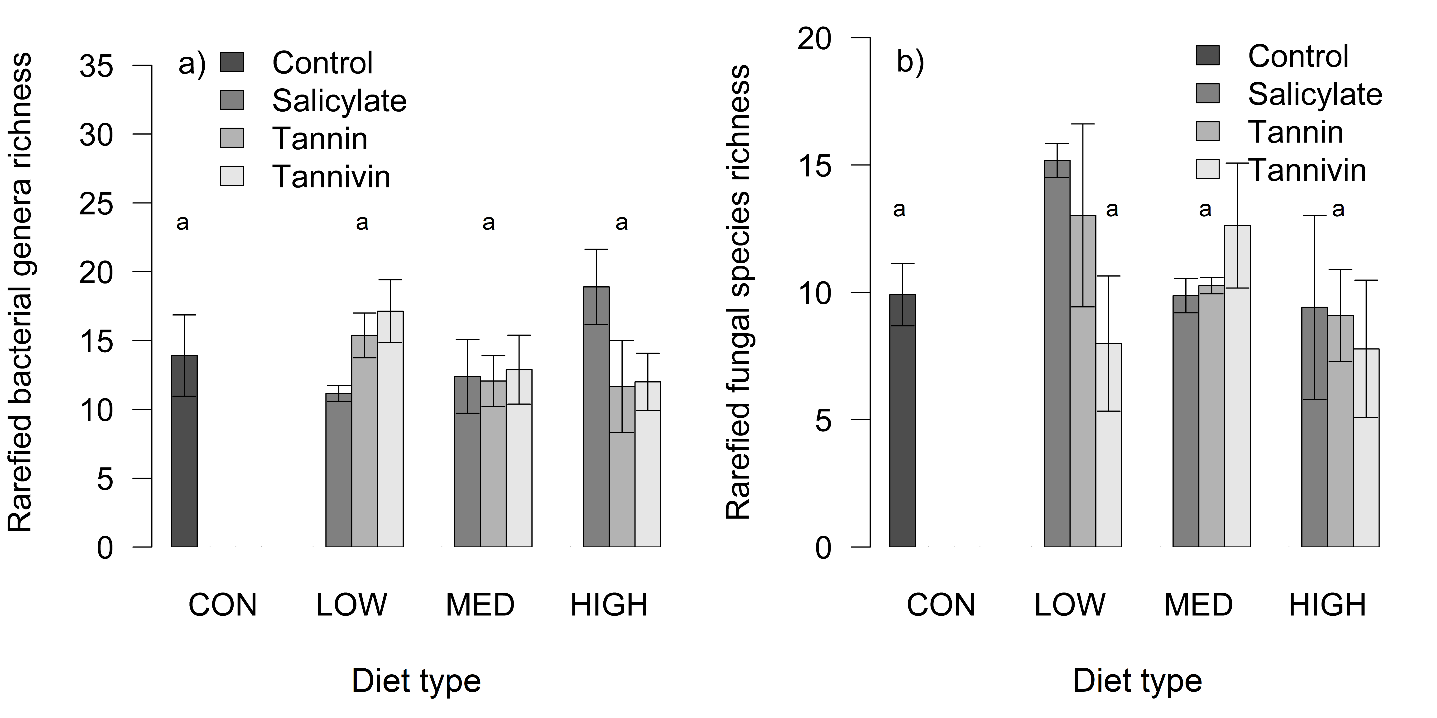


**Fig. S7.** Redundancy analysis (RDA) plot showing similarity in the composition of the a) bacterial microbiota of artificial diet (df = 27, F = 1.30, P = 0.177); b) fungal microbiota of artificial diet (df = 27, F =3.00, P = 0.006).


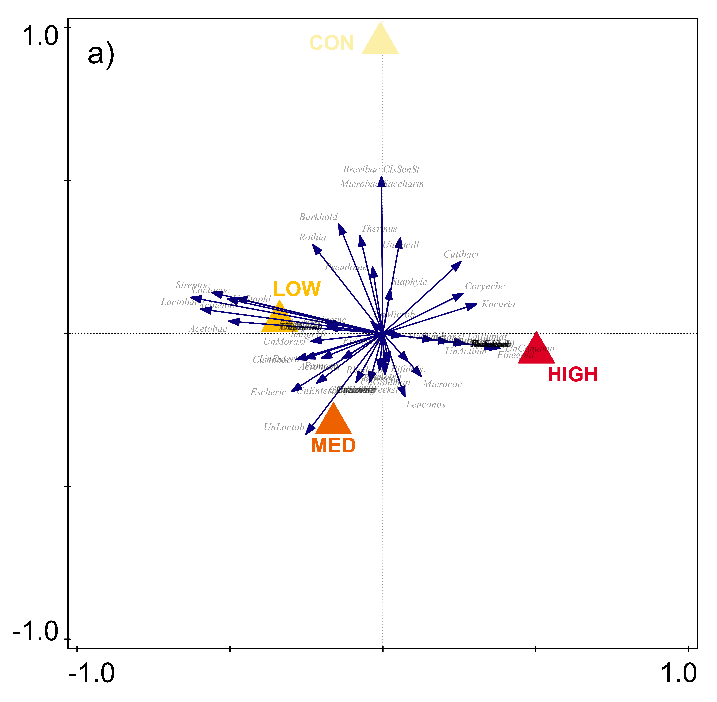

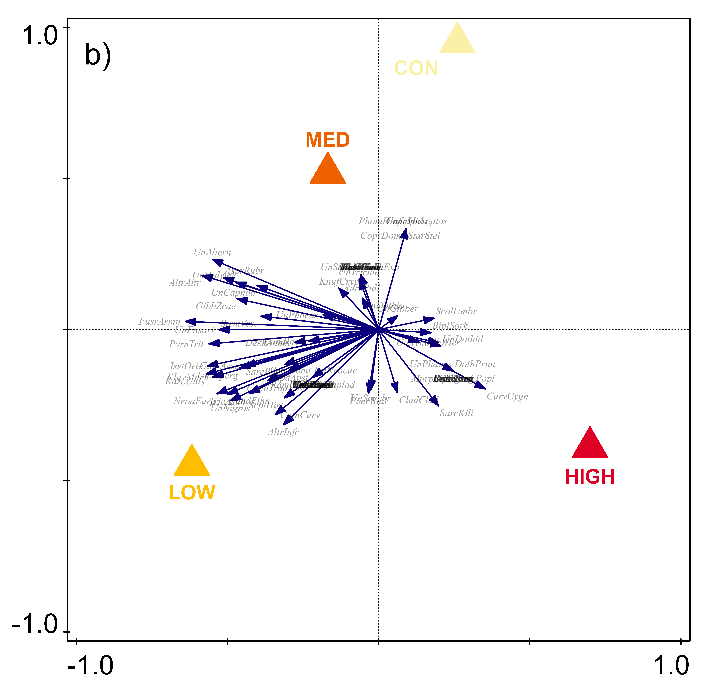


**Fig S8**. Comparison of the fit of the neutral community model (NCM) of a) bacterial and b) fungal community assembly for leaf-fed larvae. Solid blue lines indicate the best fit to the NCM according to Sloan et al. (64), and the dashed blue lines represent 95% confidence intervals (CIs) around the model prediction. Bacterial genera/ fungal species that occur more or less frequently than predicted by the NCM are shown in different colors. m = metacommunity size times immigration; r^2^ = the fit to this model


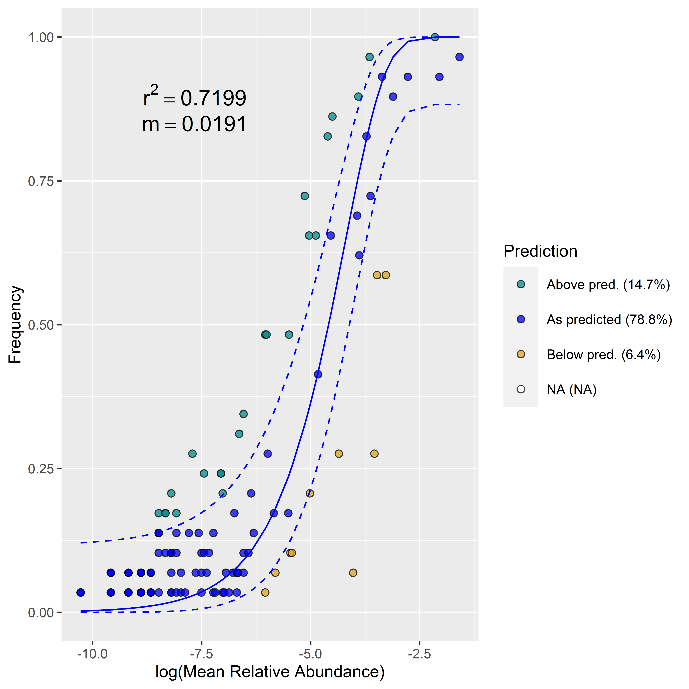

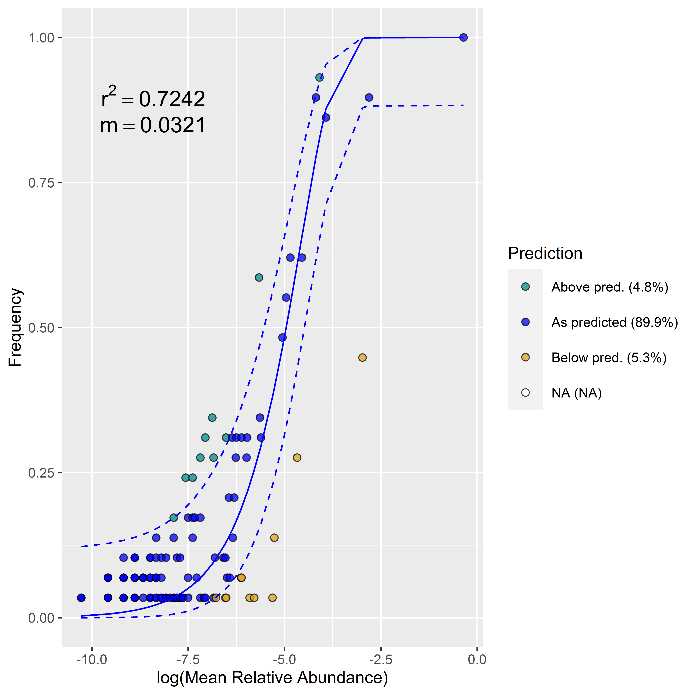


**Fig. S9.** Top 10 selected bacterial genera (frequency ≥ 25) with a significant trend (P < 0.05) of increasing or decreasing proportion of reads in increasing PSM concentration level (mean ± SE).


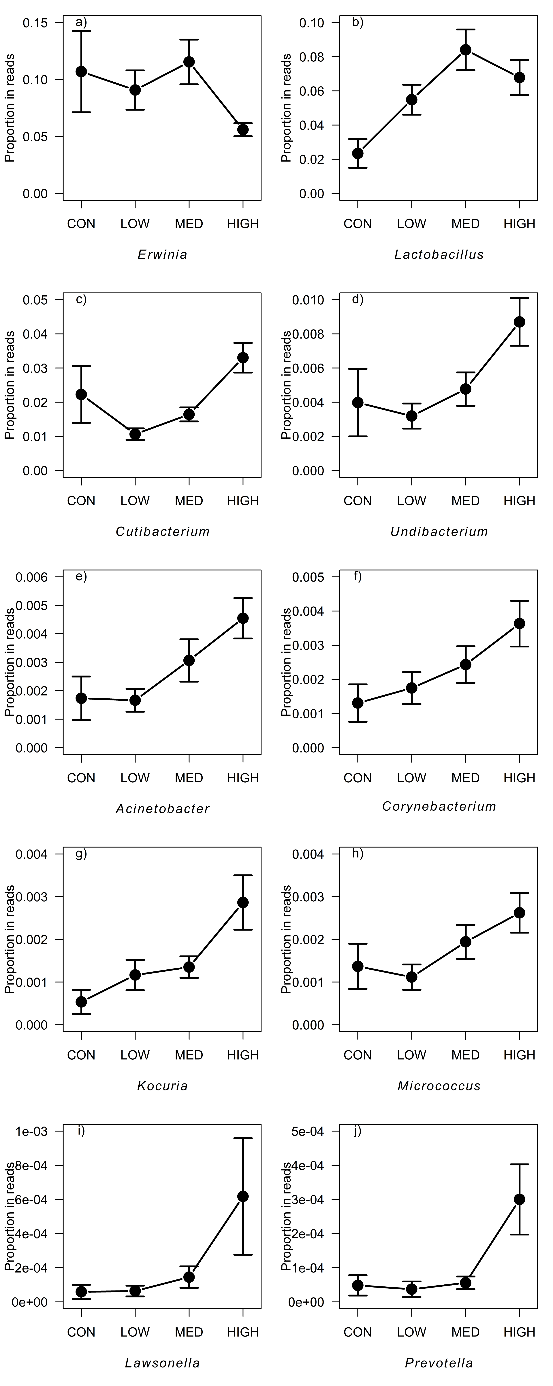


**Fig. S10.** Top four selected fungal taxa (determined at least to the genus level, frequency ≥ 25) with a significant trend (P < 0.05) of increasing or decreasing proportion of reads in increasing PSMs concentration level (mean ± SE).


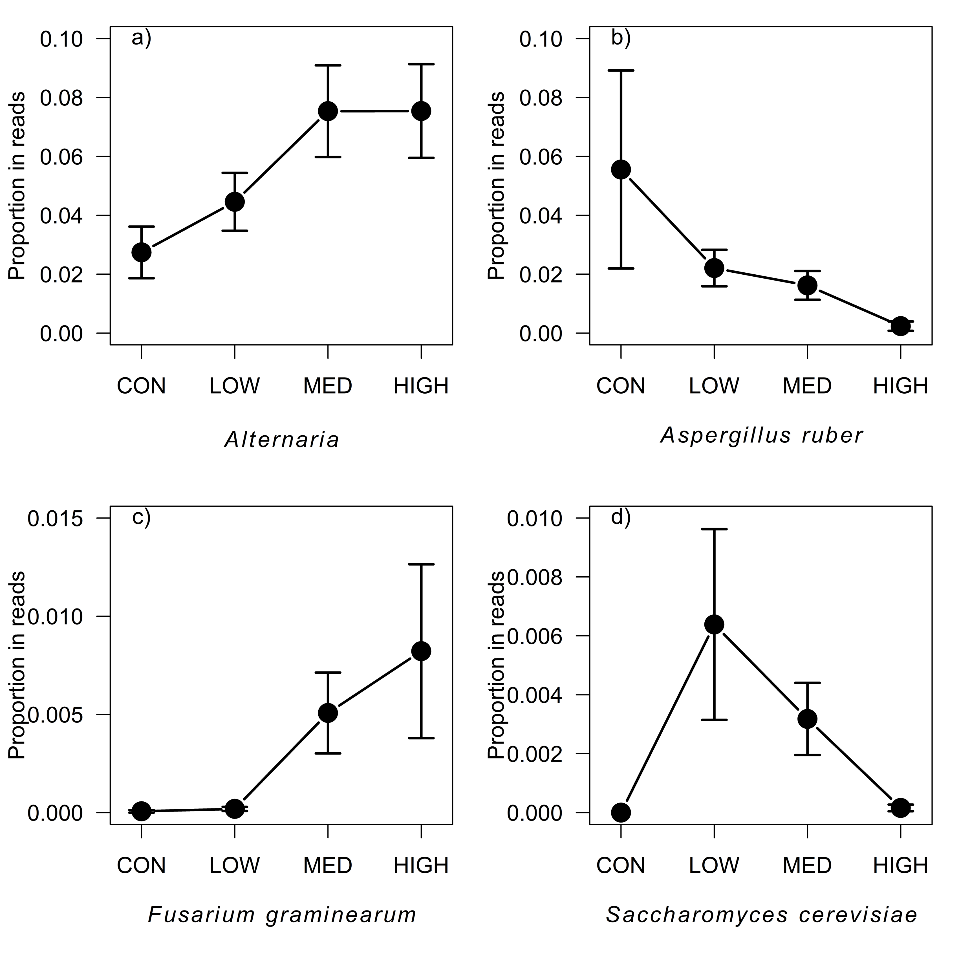


**Fig. S11**. Sparse correlation networks with subplots found by the walk trap algorithm for a) leaf-fed larvae, b) starving larvae, c) AD-fed larvae on the control diet, d) low PSMs-concentration AD-fed larvae, e) medium PSMs-concentration AD-fed larvae, f) high PSMs-concentration AD-fed larvae. Different colors represent different subplots (communities), and hub taxa are circled with a double line. Network analysis was done and plots were created using the libraries ‘MCL’ (1), ‘SpiecEasi’ (2), ‘igraph’ (3), and ‘qgraph’ (4).


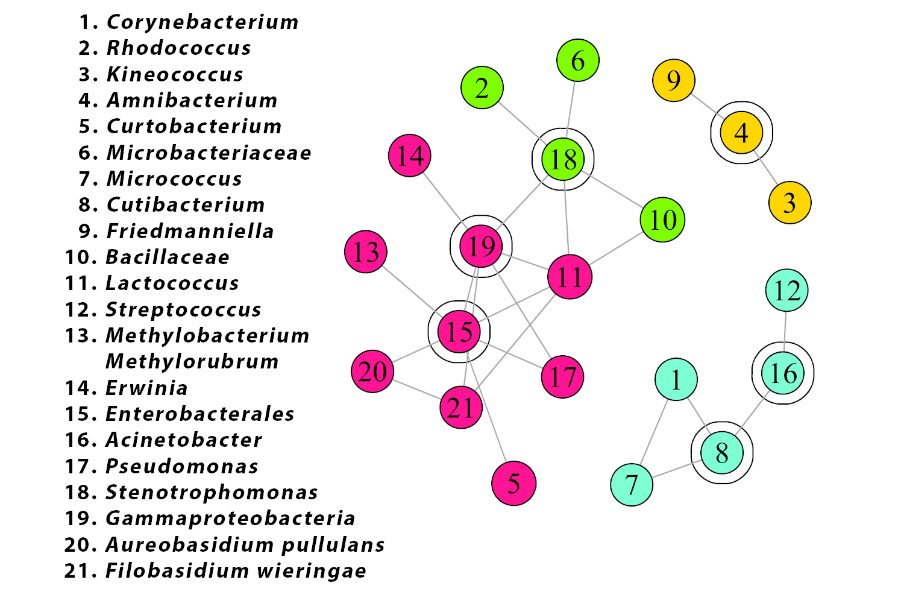


a)


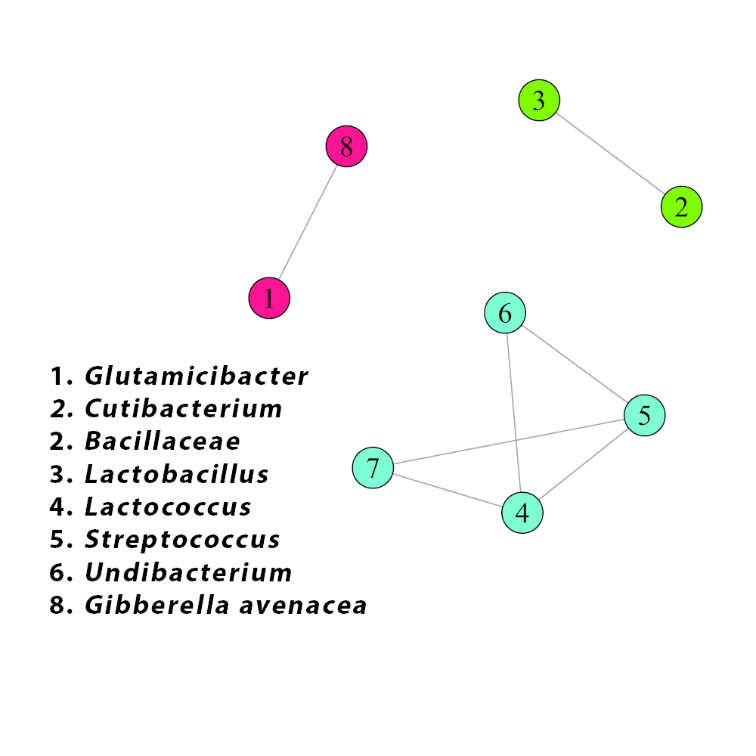

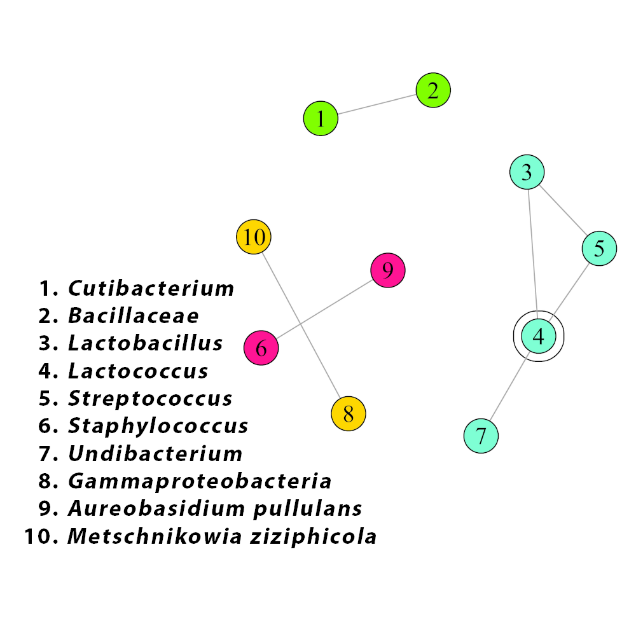


b)

c)


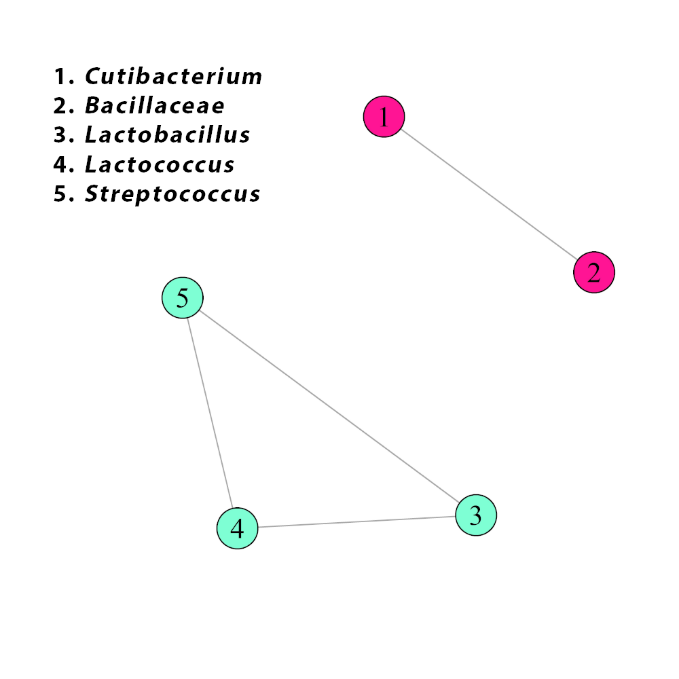

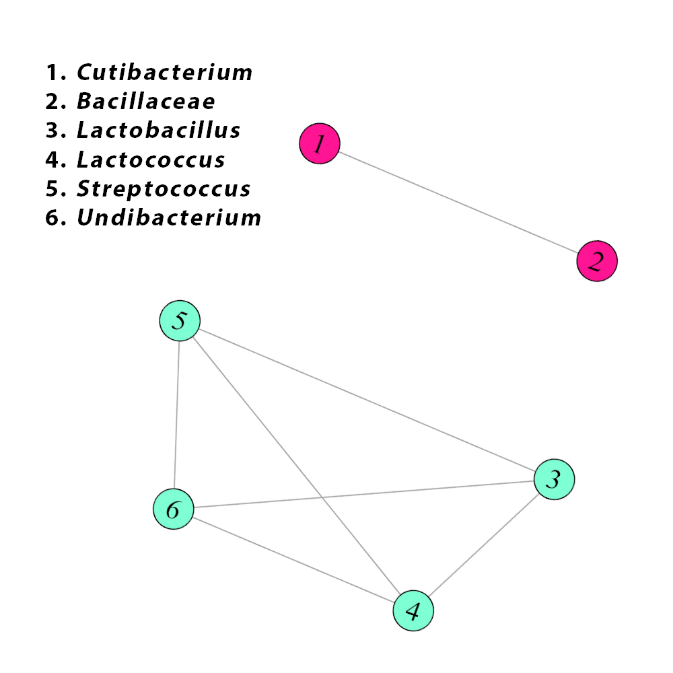


e)

d)


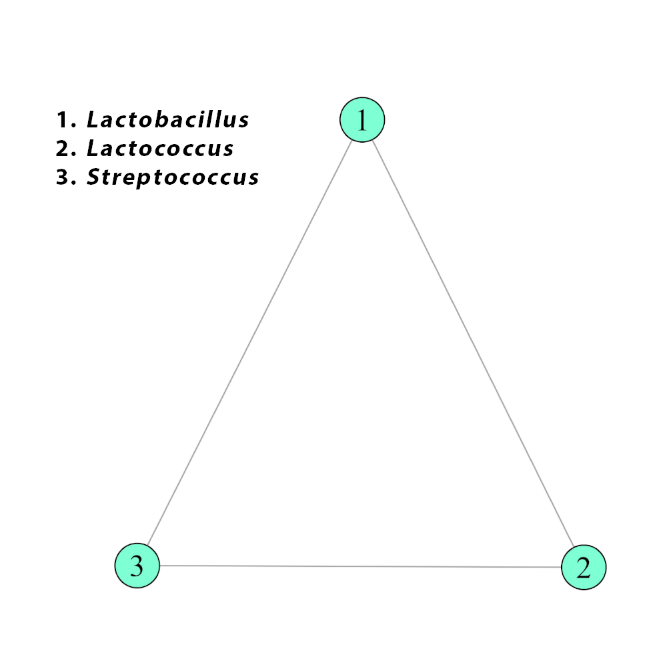


f)

**Table S1.** List of caterpillar species used in the experiment with number of individuals fed on artificial diet treated with various types of plant secondary metabolites (PSMs; tannic acid, tannivin, salicylic acid) and their concentrations (low, medium, high). Three types of controls included caterpillars fed on (1) artificial diet without PSMs (no PSMs), (2) caterpillars fed on leaves from the field (leaf-fed), and (3) caterpillars without any food provided during the experiment (starved).

|  |  |  | **Tannic acid** | | | | | **Tannivin** | | | | | | | **Salicylic acid** | | | | | | **Control** | | | |  | | |
| --- | --- | --- | --- | --- | --- | --- | --- | --- | --- | --- | --- | --- | --- | --- | --- | --- | --- | --- | --- | --- | --- | --- | --- | --- | --- | --- | --- |
| **Species** | **Family** | **Diet width** | low | medium | | high | | | low | | medium | | high | | low | | medium | | high | | | no PSMs | leaf-fed | Starved | **Σ** |  |  |
| *Lymantria dispar* | Erebidae | polyphagous | 4 | | 4 | | 4 | | | 4 | | 4 | | 4 | | 4 | | 4 | | 4 | | 4 | 5 | 3 | 48 | |  |
| *Agriopis aurantiaria* | Geometridae | polyphagous | 4 | | 4 | | 4 | | | 4 | | 4 | | 4 | | 3 | | 4 | | 4 | | 4 | 5 | 4 | 48 | |  |
| *Erannis defoliaria* | Geometridae | polyphagous | 4 | | 4 | | 4 | | | 4 | | 4 | | 4 | | 4 | | 4 | | 4 | | 4 | 5 | 4 | 49 | |  |
| *Phigalia pilosaria* | Geometridae | polyphagous | 3 | | 3 | | 4 | | | 2 | | 3 | | 4 | | 4 | | 3 | | 3 | | 2 | 5 | 4 | 40 | |  |
| *Eupsilia transversa* | Noctuidae | polyphagous | 4 | | 4 | | 4 | | | 4 | | 5 | | 4 | | 4 | | 4 | | 4 | | 4 | 3 | 4 | 48 | |  |
| *Orthosia cruda* | Noctuidae | polyphagous | 4 | | 4 | | 4 | | | 4 | | 4 | | 4 | | 4 | | 4 | | 4 | | 4 | 5 | 4 | 49 | |  |
| *Orthosia miniosa* | Noctuidae | polyphagous | 4 | | 4 | | 4 | | | 4 | | 4 | | 4 | | 4 | | 4 | | 4 | | 4 | 3 | 4 | 47 | |  |
| *Catocala sponsa* | Erebidae | monophagous | 5 | | 5 | | 4 | | | 5 | | 5 | | 4 | | 4 | | 2 | | 2 | | 6 | 3 | 3 | 48 | |  |
| *Hypaurotis quercus* | Lycaenidae | monophagous | 2 | | 5 | | 5 | | | 3 | | 1 | | 3 | | 3 | | 2 | | 3 | | 1 | 5 | 3 | 36 | |  |
|  |  | **Σ** | 34 | | 37 | | 37 | | | 34 | | 34 | | 35 | | 34 | | 31 | | 32 | | 33 | 39 | 33 | 413 | |  |

**Table S4.** Results of qPCR analysis. Mean and median of bacterial and fungal loads in caterpillar guts (number of cells/g of gut tissue) across different treatment types (PSM types: salicylic acid, tannic acid, tannivin; Control: caterpillars fed by leaves, fed by AD without PSM or starved individuals) and PSM concentration (low, medium or high)

|  |  | **Bacteria (mean)** | **Bacteria (median)** | **Fungi (mean)** | **Fungi (median)** |
| --- | --- | --- | --- | --- | --- |
| Treatment type |  |  |  |  |  |
|  | salicylic acid | 1,945,062 | 313,324 | 5,748 | 45 |
| tannic acid | 2,201,602 | 421,231 | 68 | 18 |  |
| tannivin | 553,862 | 150,249 | 76 | 29 |  |
| leaf-fed | 2,533,422 | 1,853,831 | 4,161 | 2,430 |  |
| starved | 10,322,427 | 1,137,388 | 282 | 285 |  |
| without PSM | 79,814 | 73,781 | 37 | 31 |  |
| PSM concentration |  |  |  |  |  |
|  | low | 1,089,946 | 482,166 | 68 | 38 |
| medium | 287,484 | 184,010 | 5,754 | 29 |  |
| high | 3,323,096 | 421,231 | 69 | 10 |  |

**Table S5**. Fit of the neutral community model (NCM) of bacterial community assembly. **Type**: The groups of design for which the model was fitted. **Above**: Number of bacterial genera that occur more frequently than predicted by the NCM; **As pred**: Number of bacterial genera fitting the model; **Below**: Number of bacterial genera that occur less frequently than predicted by the NCM; **Out pred**: Above + Below; **As pred %**: Percentage of bacterial genera fitting the model; **Out pred**: Percentage of bacterial genera non-fitting the model.

| Type | Above | As pred | Below | Out pred | As pred % | Out pred % |
| --- | --- | --- | --- | --- | --- | --- |
| Control AD | 14 | 93 | 4 | 18 | 83.78 | 16.22 |
| Starved | 14 | 89 | 2 | 16 | 84.76 | 15.24 |
| Leaf-fed | 23 | 123 | 10 | 33 | 78.85 | 21.15 |
| Salicylate – High | 13 | 123 | 4 | 17 | 87.86 | 12.14 |
| Salicylate – Medium | 18 | 139 | 9 | 27 | 83.73 | 16.27 |
| Salicylate – Low | 13 | 68 | 5 | 18 | 79.07 | 20.93 |
| Tannic Acid – High | 7 | 70 | 6 | 13 | 84.34 | 15.66 |
| Tannic Acid – Medium | 11 | 65 | 3 | 14 | 82.28 | 17.72 |
| Tannic Acid – Low | 9 | 64 | 6 | 15 | 81.01 | 18.99 |
| Tannivin – High | 17 | 104 | 4 | 21 | 83.20 | 16.80 |
| Tannivin – Medium | 15 | 76 | 7 | 22 | 77.55 | 22.45 |
| Tannivin – High | 18 | 87 | 5 | 23 | 79.09 | 20.91 |
| Artificial diet | 4 | 51 | 3 | 7 | 87.93 | 12.07 |

**Table S6**. Fit of the neutral community model (NCM) of fungal community assembly. **Type**: The groups of design for which the model was fitted. **Above**: Number of fungal species that occur more frequently than predicted by the NCM; **As pred**: Number of fungal species fitting the model; **Below**: Number of fungal species that occur less frequently than predicted by the NCM; **Out pred**: Above + Below; **As pred %**: Percentage of fungal species fitting the model; **Out pred**: Percentage of fungal species non-fitting the model.

| Type | Above | As pred | Below | Out pred | As pred % | Out pred % |
| --- | --- | --- | --- | --- | --- | --- |
| Control AD | 9 | 95 | 5 | 14 | 87.16 | 12.84 |
| Starved | 13 | 86 | 3 | 16 | 84.31 | 15.69 |
| Leaf-fed | 10 | 187 | 11 | 21 | 89.90 | 10.10 |
| Salicylate – High | 4 | 87 | 4 | 8 | 91.58 | 8.42 |
| Salicylate – Medium | 3 | 65 | 0 | 3 | 95.59 | 4.41 |
| Salicylate – Low | 4 | 87 | 1 | 5 | 94.57 | 5.43 |
| Tannic Acid – High | 5 | 61 | 1 | 6 | 91.04 | 8.96 |
| Tannic Acid –Medium | 2 | 68 | 3 | 5 | 93.15 | 6.85 |
| Tannic Acid – Low | 3 | 53 | 1 | 4 | 92.98 | 7.02 |
| Tannivin – High | 1 | 62 | 2 | 3 | 95.38 | 4.62 |
| Tannivin – Medium | 6 | 76 | 1 | 7 | 91.57 | 8.43 |
| Tannivin – High | 4 | 28 | 0 | 4 | 87.50 | 12.50 |
| Artificial diet | 5 | 46 | 3 | 8 | 85.19 | 14.81 |

**Table S7**. The fit of power-law distribution function from network analysis. **Group**: Group of design of the experiment (Leaf-fed, Starved, Control AD, low – Low PSMs concentration, Med – medium PSMs concentration, High – high PSMs concentration, and Artificial diet); **α**: the exponent of the fitted power-law distribution; **logLik**: log-likelihood of the fitted parameters; **D**: test statistic of a Kolmogorov-Smirnov test; **p-value**: p-value of the hypothesis that the original data could have been drawn from the fitted power-law distribution.

| Group | α | logLik | D | p-value |
| --- | --- | --- | --- | --- |
| Leaf-fed | 1.88 | -41.26 | 0.15 | 0.670 |
| Starved | 2.16 | -11.07 | 0.16 | 0.986 |
| Control diet | 2.50 | -7.84 | 0.12 | 1.000 |
| Low | 2.22 | -6.57 | 0.28 | 0.842 |
| Med | 1.85 | -11.70 | 0.37 | 0.383 |
| High | 1.88 | -5.63 | 0.56 | 0.297 |
| Artificial diet | 1.95 | -24.02 | 0.17 | 0.797 |

**Table S8**. The fit of power-law distribution function from network analysis on the species level. **Species**: larval species; **α**: the exponent of the fitted power-law distribution; **logLik**: log-likelihood of the fitted parameters; **D**: test statistic of a Kolmogorov-Smirnov test; **p-value**: p-value of the hypothesis that the original data could have been drawn from the fitted power-law distribution

| Species | α | logLik | D | p-value |
| --- | --- | --- | --- | --- |
| *Agriopis aurantiaria* | 2.22 | -6.57 | 0.28 | 0.842 |
| *Catocala sponsa* | 1.88 | -5.63 | 0.56 | 0.297 |
| *Erannis defoliaria* | 1.88 | -5.63 | 0.56 | 0.297 |
| *Eupsilia transversa* | 2.27 | -12.50 | 0.19 | 0.863 |
| *Hypaurotis quercus* | 2.31 | -14.42 | 0.18 | 0.853 |
| *Lymantria dispar* | 2.43 | -11.89 | 0.18 | 0.849 |
| *Orthosia cruda* | 1.88 | -5.63 | 0.56 | 0.297 |
| *Orthosia miniosa* | 2.35 | -9.25 | 0.21 | 0.866 |
| *Phigalia pilosaria* | 2.22 | -6.57 | 0.28 | 0.842 |

**References**

1. Jäger ML. 2015. MCL: Markov Cluster Algorithm. R package version 1.0.

2. Kurtz Z, Mueller C, Miraldi E, Bonneau R. 2023. SpiecEasi: Sparse Inverse Covariance for Ecological Statistical Inference. R package version 1.1.2.

3. Csardi G, Nepusz T. 2006. The igraph software package for complex network research. InterJournal Complex Syst.

4. Epskamp S, Cramer AOJ, Waldorp LJ, Schmittmann VD, Borsboom D. 2012. qgraph: Network Visualizations of Relationships in Psychometric Data. J Stat Softw 48:1–18.
